# Trends in antibiotic use among outpatients in New Delhi, India

**DOI:** 10.1186/1471-2334-11-99

**Published:** 2011-04-20

**Authors:** Anita Kotwani, Kathleen Holloway

**Affiliations:** 1 Department of Pharmacology, V. P. Chest Institute, University of Delhi, Delhi, India; 2 Essential Drugs and Other Medicines, World Health Organization, Regional Office for South East Asia, New Delhi, India

## Abstract

**Background:**

The overall volume of antibiotic consumption in the community is one of the foremost causes of antimicrobial resistance. There is much ad-hoc information about the inappropriate consumption of antibiotics, over-the-counter availability, and inadequate dosage but there is very little actual evidence of community practices.

**Methods:**

This study surveyed antibiotic use in the community (December 2007-November 2008) using the established methodology of patient exit interviews at three types of facilities: 20 private retail pharmacies, 10 public sector facilities, and 20 private clinics to obtain a complete picture of community antibiotic use over a year. The Anatomical Therapeutic Chemical (ATC) classification and the Defined Daily Dose (DDD) measurement units were assigned to the data. Antibiotic use was measured as DDD/1000 patients visiting the facility and also as percent of patients receiving an antibiotic.

**Results:**

During the data collection period, 17995, 9205, and 5922 patients visiting private retail pharmacies, public facilities and private clinics, respectively, were included in our study. 39% of the patients attending private retail pharmacies and public facilities and 43% of patients visiting private clinics were prescribed at least one antibiotic. Consumption patterns of antibiotics were similar at private retail pharmacies and private clinics where fluoroquinolones, cephalosporins, and extended spectrum penicillins were the three most commonly prescribed groups of antibiotics. At public facilities, there was a more even use of all the major antibiotic groups including penicillins, fluoroquinolones, macrolides, cephalosporins, tetracyclines, and cotrimoxazole. Newer members from each class of antibiotics were prescribed. Not much seasonal variation was seen although slightly higher consumption of some antibiotics in winter and slightly higher consumption of fluoroquinolones during the rainy season were observed.

**Conclusions:**

A very high consumption of antibiotics was observed in both public and private sector outpatients. There was a high use of broad spectrum and newer antibiotics in the community. Suitable and sustainable interventions should be implemented to promote rational use of antibiotics that will help in decreasing the menace of antibiotic resistance.

## Background

The current worldwide increase in antimicrobial resistance (AMR) and, simultaneously, the downward trend in the development of new antibiotics have serious public health and economic implications. The increased resistance is a result of many factors, but the foremost cause is the overall volume of antibiotic consumption. About 80% of antibiotics are used in the community and the rest are used in hospitals [1,2]. It is estimated that 20-50% of all antibiotics use is inappropriate, resulting in an increased risk of side effects, higher costs and higher rates of AMR in community pathogens [3]. Detailed surveillance of antibiotic use in the community is one strategy to guide and control antibiotic overuse and misuse. In a number of developed countries, extensive surveillance programmes have been developed to study patterns of AMR and antibiotic use [4-6]. However, the problem of AMR has received relatively little recognition in developing countries and the ability to undertake extensive surveillance is lacking in resource-constrained settings. Thus, there is a lack of community-based databases on AMR and antibiotic use in developing countries. In developing countries antibiotics can be obtained easily from private retail pharmacies without prescription and pharmacists also advise and dispense antibiotics to patients [7].

In collaboration with the World Health Organization (WHO) a pilot project was conducted in New Delhi, India (2002-2005) [8] and elsewhere [9] to develop validated reproducible and sustainable surveillance methodologies to quantify antimicrobial resistance and antibiotic use in the community. The pilot project conducted by us in New Delhi, India, utilized the same methodology as a previous study that monitored antibiotic use in the community through patient exit interviews at private retail pharmacies [8]. This study, conducted during December 2007 - November 2008, expanded the established methodology of exiting patient interviews to a detailed community surveillance of antibiotic use in three types of facilities: private retail pharmacies, public sector facilities, and private clinics. The primary aim of this study was to determine the pattern and consumption of antibiotics at the community level in the public and private sectors over one year.

## Methods

Surveillance of antibiotic use was done by collecting data from four municipal wards (residential localities) of New Delhi, India. The study was done in conjunction with another study (not described here) to measure the antimicrobial resistance pattern for the OPD patients of a private tertiary care hospital located in West Delhi. Hence, the antibiotic use data was collected from four municipal wards around this hospital - the same 4 wards as used in the previous study in private retail pharmacies [8]. The four areas were - Rajinder Nagar, Patel Nagar, Karol Bagh and Rajouri Garden.

### Settings and Facility selection

Both the public and private sectors were included in the survey. Medicines are provided free of charge to patients in public sector facilities but must be paid for in private sector facilities. In order to get a complete picture of antibiotic use in the community, three different types of facilities were chosen:

1. Public sector - All 10 facilities under the Government of National Capital Territory of Delhi (GNCT Delhi) in our catchment area were enrolled for the survey. The facilities were 8 dispensaries (Primary care facility) and 2 hospitals (Secondary care level);

2. Private retail pharmacies - A total of 185 licensed private retail shops were located in the four chosen municipal wards from which 20 well-stocked, private retail shops, willing to cooperate, were chosen for the study;

3. A total of 20 private sector doctors, who were willing to cooperate for the study and were practicing in the chosen areas, were chosen and consisted of 4 paediatricians, 3 physicians, 12 general practitioners (GPs), and 1 dermatologist (who was also practicing as GP).

A convenience sample for private sector facilities was used because many doctors and retail pharmacy shops do not tolerate continued data collection processes. Moreover we wanted to include facilities with a sufficient number of patients per day. A good liaison was maintained with all the participants and their professional associations, who helped in enrolling the facilities throughout the study period.

### Data collection methodology: Patient exit interviews

Trained data collectors (pharmacists) collected antibiotic use data by conducting exiting interviews with all patients receiving/purchasing an antibiotic on leaving the facility. For private retail pharmacies any patient purchasing an antibiotic, whether with a prescription, without a prescription, or on the advice of chemist, was interviewed. For public sector facilities and private clinics all the patients interviewed were out-patients. In private facilities, exiting patient interviews were conducted in a part of the waiting room after the patient had left the consultation room. In pharmacies, exiting patient interviews were conducted in a part of the shop or on the street outside. At public facilities, exit interviews were conducted near the pharmacy of the same facility. A pre-designed proforma was used to collect data regarding the name of the antibiotic, the strength, the dose and the number of units prescribed by the doctor, and the number of units dispensed or purchased.

### Sample size

30 exit interviews were conducted in each public sector facility and private retail pharmacy in each month. However, only about 10-12 exit interviews were conducted in each private clinic every month as the patient flow was much less and it was not possible to wait the required time to collect the same number of patients due to resource constraints. The rationale for conducting this number of exit interviews is from the WHO manual, "How to investigate drug use in health facilities" which recommends 600 encounters (20 facilities and 30 patients/prescriptions per facility) for one survey [10]. Data collectors' schedules were randomly prepared for the day and time (two hour) of each visit. During each facility visit, all patients receiving any antibiotic were interviewed. Also, the total number of patients visiting each facility (whether or not they received antibiotics) during the time taken to collect the desired number of antibiotic-containing prescriptions was counted by a second data collector and recorded at each visit. Data were collected monthly from the same facilities in all three types of facility throughout the study period. Each facility had to be visited 3-5 times per month to collect the required number of exit interviews/prescriptions.

### Outcome measures

The Anatomical Therapeutic Chemical (ATC) classification and the Defined Daily Dose (DDD), ATC/DDD (2006), measurement units were assigned to the data [11]. Consumption of antibiotics was expressed in two ways - the total number of DDDs/1000 patients attending the facility and the percentage of patients receiving an antibiotic. To calculate the total DDDs for each exit interview, the strength of dosage form (tablet/capsule etc.) was multiplied by the total units (number of tablets/capsules) of each antibiotic received and the resulting figure then divided by the DDD of that antibiotic to give the total DDDs that the patient received. Antibiotics for local use, like creams and drops, were not included for calculating the DDDs consumed. The denominator for exit interview data was the number of patients attending the facilities (whether or not they received antibiotics) during the time taken to do the target number of exit interviews for patients receiving antibiotics. Consumption in terms of DDDs per population per day was not used since complete data (covering all patient attendees) could not be collected from any facility nor could all private facilities in the concerned areas be included.

### Data management

All the data collected was entered into software developed in Visual Basic, SQL Server and Crystal Reports. The same software was used to analyse the data.

### Ethical Approval

Ethical approval for the study was obtained from Vallabhbhai Patel Chest Institute, University of Delhi, India and also from WHO Ethics Review Committee. Informed consent was obtained from all participants and facilities involved in the study.

## Results

### Antibiotic Use in the Community in Different Sectors

In enrolled private retail pharmacies, 7101 patients (39.5%) out of 17995 patients visiting the pharmacies during data collection purchased an antibiotic (and were thus interviewed). In enrolled public facilities, 3615 (39.3%) out of 9205 patients were prescribed an antibiotic. In enrolled private clinics, 2571 (43.4%) out of 5922 patients were prescribed an antibiotic.

### Annual use and consumption of antibiotic

Table 1 shows annual use measured as the percent of prescriptions containing the major classes of antibiotics by facility type in the four areas surveyed. In private retail pharmacies and at private clinics, cephalosporins (J01DA) and fluoroquinolones (J01MA) were the most prescribed antibiotic class, followed by the penicillins (J01C), while the older antibiotics such as cotrimoxazole (J01EE01) and the tetracyclines (J01A), were infrequently used. By contrast, in public sector facilities, all the groups of antibiotics were used - penicillins (10.2%), fluroquinolones (9.0%), macrolides (6.6%), cephalosporins (5.7%,) cotrimoxazole (4.4%) and tetracyclines (3.4% ) - in all the areas surveyed.

**Table 1:** Percent of prescriptions with various classes of antibiotics in the facilities surveyed in Delhi, India (December 2007-November 2008)

Antibiotics	Public sector	Private retail pharmacies	Private clinics
Cephalosporins (J01DA)	5.7%	14.4%	11.6%

Flouroquinolones (J01MA)	9.0%	11.1%	13.7%

Penicillins (J01C)	10.2%	8.8%	8.7%

Macrolides (J01FA)	6.6%	4.0%	3.8%

Tetracyclines (J01A)	3.4%	0.6%	2.2%

Co-trimoxazole (J01EE01)	4.4%	0.3%	2.6%

Aminoglycosides (J01G)	-	0.3%	0.8%

**Total**	**39.3%**	**39.5%**	**43.4%**

Table 2 shows annual consumption of various classes of antibiotics measured as DDD/1000 patients by facility type. Consumption measured in terms of DDD/1000 patients showed similar trends as when measured in terms of percentage of patients receiving an antibiotic. Thus, for both private pharmacies and private clinics, highest consumption was for the fluoroquinolones, closely followed by the cephalosporins and the penicillins. In the public sector, highest consumption was of penicillins and fluoroquinolones, followed by the macrolides, tetracyclines, and cephalosporins. Macrolides (J01FA) and tetracyclines were not consumed in large quantities in the private sector. Cotrimoxazole was the least consumed antimicrobial in both the public and private sectors (Table 2). The pattern of consumption of various groups of the antibiotics appeared similar in all the four areas studied.

**Table 2:** Antibiotic use (DDD/ 1000 patients) in the facilities surveyed in Delhi, India (December 2007-November 2008)

Antibiotic name	Public sector		Private retail		Private clinic	
	DDD	%*	DDD	%*	DDD	%*
**Cephalosporins J01DA**						
Cefuroxime	3132	7.2%	13511	10.8%	8987	11.0%
Cephalexin	971	2.2%				
Cefixime	-		8065	6.4%	5919	7.3%
Cefixime + clavulanic acid	-		4127	3.3%	2897	3.5%
Others	31		7256		3635	
Subtotal	**4134**		**32959**		**21929**	

**Fluoroquinolones J01MA**						
Ofloxacin	5516	12.7%	15652	12.5%	13222	16.2%
Ciprofloxacin	4367	10.1%	7557	6.0%	5434	6.7%
Levofloxacin	-		5559	4.4%	4586	5.6%
Norfloxacin	2590	6.0%	3332	2.6%	1028	1.3%
Others	86		5639		2750	
Subtotal	**12559**		**37739**		**27020**	

**Penicillins J01C**						
Amoxicillin	6403	14.7%	8240	6.6%	2263	2.8%
Amoxicillin+Calvulinic acid	2873	6.6%	16299	13.0%	14370	17.6%
Amoxicillin+Cloxacillin	-		4082	3.2%	3065	3.8%
Ampicillin	2234	5.1%				
Others	1078		1859		148	
Subtotal	**12588**		**30480**		**19846**	

**Macrolides J01FA**						
Roxithromycin	3995	9.2%	6836	5.4%	3353	4.1%
Azithromycin	38	0.09%	5404	4.3%	3648	4.5%
Erythromycin	2286	5.3%	2535	2.0%	292	0.3%
Others	0		801		719	
Subtotal	**6319**		**15576**		**8012**	

**Tetracycline J01A**						
Doxicycline	4210	9.7%	6147	4.9%	4440	5.4%
Tetracycline	1759	4.0%	949	0.8%	7	0.008%
Others	15		12		33	
Subtotal	**5884**		**7108**		**4480**	

**Combinations of Sulfonamide with Trimethoprim (J01EE)**						
Cotriamoxazole	**1806**	4.0%	**1682**	1.3%	**180**	0.2%

**Total DDDs/1000 patients for all antibiotics**	**43390**		**125544**		**81467**	

*% of total DDDs/1000 patients for all antibiotics

### Pattern of consumption of members from different classes of antibiotics

Table 2 shows the top three most commonly prescribed members from each class of antibiotic and the percentage for each of these antibiotics out of the total DDDs/1000 patients' consumption of all antibiotics. Patterns of use in private sector, at both retail pharmacies and private clinics, were similar. Newer antibiotics were often used more than the older ones. Thus, cefuroxime, cefixime and cefixime+clavulanic acid were the most commonly prescribed cephalosporins and ofloxacin, ciprofloxacin, levofloxacin, and norfloxacin were the most commonly prescribed fluoroquinolones. For penicillins, only the extended spectrum penicillins were prescribed, viz. amoxicillin+clavulanic acid, amoxicillin, and amoxicillin+cloxacillin. For macrolides, roxithromycin and azithromycin were prescribed/purchased more than erythromycin.

At public facilities, while the newer members from each class of antibiotic were also used, there was more use of the older antibiotics than in the private sector. Thus, newer antibiotics such as cefuroxime, ofloxacin, and roxithromycin were commonly used as in the private sector but older antibiotics such as cephalexin, erythromycin, cotrimoxazole, and tetracyclines were also commonly used. However, older antibiotics were used sparingly in the private sector (Table 2). For penicillins, in the public sector, the highest prescribed member was amoxicillin followed by amoxicillin+clavulanic acid and ampicillin, whereas in the private sector, highest prescribed penicillin was amoxicillin+clavulanic acid and ampicillin was not prescribed.

### Monthly trends in the use of antibiotics

**Figure 1:**
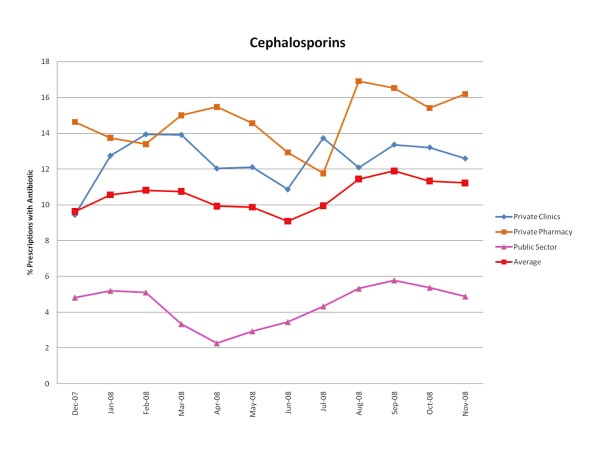
**Monthly use of cephalosporins in the community from December 2007-November 2008**.

**Figure 2:**
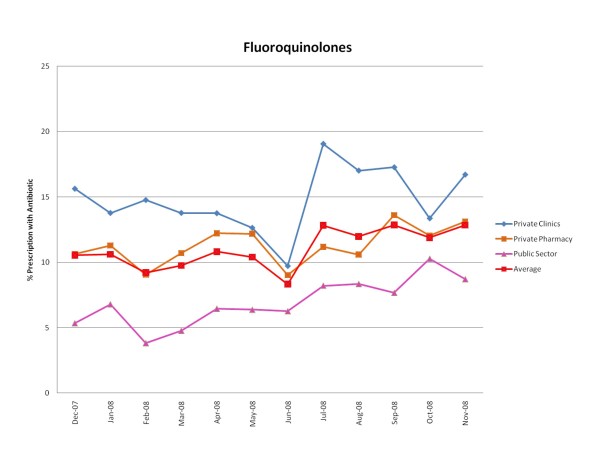
**Monthly use of fluoroquinolones in the community from December 2007-November 2008**.

**Figure 3:**
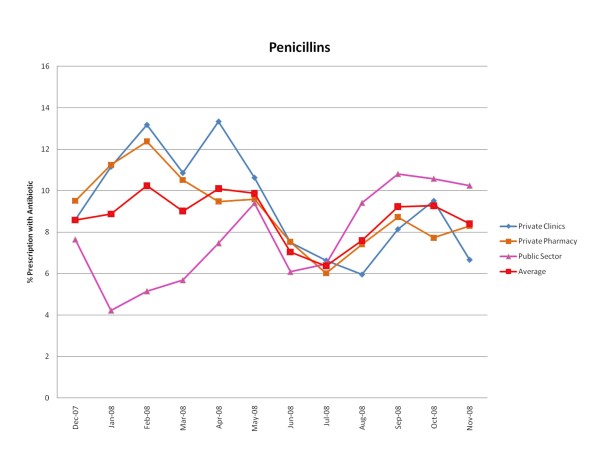
**Monthly use of penicillins in the community from December 2007-November 2008**.

**Figure 4:**
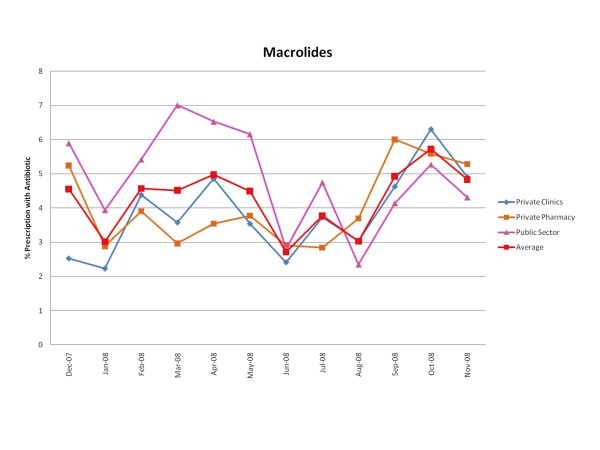
**Monthly use of macrolides in the community from December 2007-November 2008**.

**Figure 5:**
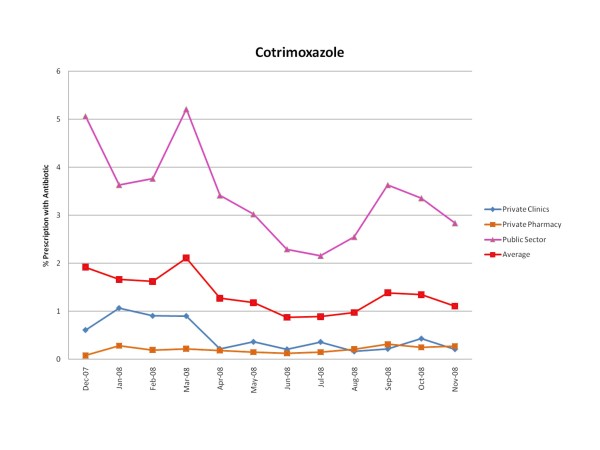
**Monthly use of cotrimoxazole in the community from December 2007-November 2008**.

**Figure 6:**
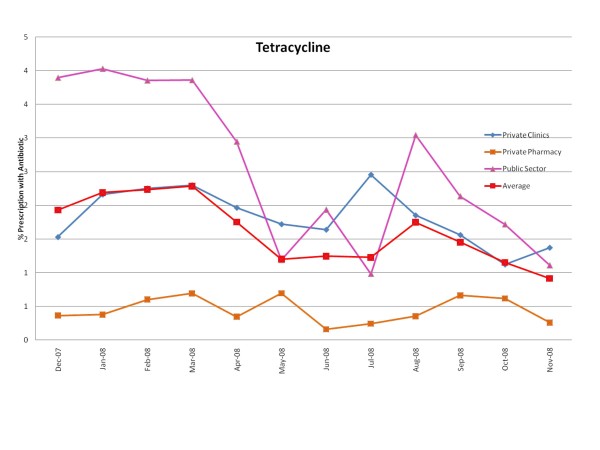
**Monthly use of tetracyclines in the community from December 2007-November 2008**.

Figures 1, 2, 3, 4, 5 and 6 show the monthly trends in the percent of prescriptions containing various classes of antibiotics in the three types of facility surveyed as well as the average consumption (across all facility types) of each antibiotic group over a year. Although there were no marked differences in the consumption of each particular antibiotic class throughout the year, there did appear to be some overall increased use of macrolides, tetracyclines, cotrimoxazole and penicillins (commonly used for acute respiratory tract infection) during the winter months December-March. By contrast, the use of fluroquinolones (commonly used for diarrhea) appeared greatest during the rainy months of summer, July-August. (Figure 2).

## Discussion

This is one of the first studies from a developing country that describes a large, comprehensive surveillance of antibiotic use in three facility types over a one-year period to get a complete picture of antibiotic use in an urban community. There is evidence at the national level [12] as well as some evidence at the patient level [13] from Europe that the incidence of resistance is positively correlated with antibiotic use in the outpatient. Hence, a continuous surveillance of antibiotic use and resistance plus detailed knowledge of antibiotic use in the community are necessary to develop and implement guidelines for antibiotic use in a particular region.

Surveillance of antimicrobial use in the community for developing countries is difficult because no databases are available and antibiotics can be purchased over-the-counter [14,15]. The present study fills the gap and the methodology used can be utilized in any developing country to collect data on outpatient antibiotic use. In this study data, on average across three facility types, 41% of patients were prescribed an antibiotic in the community. Surveillance of antibiotics conducted at two other sites in India, Vellore and Mumbai, also showed 41% and 42% average consumption of antibiotics, respectively, across three facility types [9]. Previous drug utilization studies and prescription audit studies conducted in different cities of India have shown that 39% of drugs prescribed to outdoor patients in Goa, [16] and 43% in Pune [17] were antibiotics. The data available on antibiotic use in the community in India, is often fragmented, usually being collected over a few months either for a drug use indicator study or for examining treatment of a particular disease [18,19].

Measuring the percent of antibiotic containing prescriptions is an easy and more reliable method than measuring the DDD/1000 inhabitants in these settings as the data is not collected from all the facilities serving a population. However, data on DDD consumed per 1000 patients can help in providing insight into how antibiotics are used [20]. For example, our study clearly shows that while the proportion of patients receiving antibiotics is similar in the public and private sectors (Table1), the total DDDs dispensed at public sector facilities is much less than in private retail pharmacies and private clinics (Table 2). This indicates that much smaller amounts are dispensed per patient in the public versus the private sector. It was found that in the public sector antibiotics were dispensed to patients for three days and, in the case of antibiotic shortages, even fewer days than this. By contrast, longer courses were dispensed in the private sector. In a few cases the different drug use measures gave slightly different results with regard to identifying the most frequently or highest consumed antibiotic. For example, in private retail pharmacies, cephalosporins was the most frequently consumed type of antibiotic, followed by fluoroquinolones, according to the percent of patients receiving the antibiotic (Table 1) but fluoroquinolones followed by cephaolosprins according to the measure, DDDs/1000 patients (Table 2).

The surveillance system was able to track trends and patterns of antibiotic use in all the types of facilities: public facilities, private clinics and private retail pharmacies. Findings from the present survey clearly show the importance of tracking antibiotic use in different facility types as consumption of various classes of antibiotics was clearly different. In the public sector, almost all classes of antibiotics were prescribed while in the private sector virtually only the newer antibiotics were consumed. Comparison of the results of this survey in 2008 with the pilot survey conducted at the private retail pharmacies in the same areas in 2004 [8] showed that consumption of macrolides appears to have decreased while that of cephalosporins appears to have markedly increased during the period 2004 - 2008. This is worrying from the perspective of increasing resistance to the cephalosporins. Thus, this repeat survey using the same methodology in the same areas shows itself to be a good system of surveillance of antibiotic use, catching trends and patterns of use over a period of time.

No major differences in the pattern of use of the various antibiotic groups were found between the four wards/areas surveyed for any facility types nor were any major differences found with regard to the percent of patients receiving antibiotics in four areas surveyed. In each of the four areas, the percent of patient receiving an antibiotic was 38%-41% in public facilities and 39%-41% in private retail pharmacies. However, for private clinics the percent of patients who received antibiotics in Rajouri Garden was 34% and in Karol Bagh 38% as compared to Patel Nagar where it was 56% and Rajinder Nagar where it was 50%. It is difficult to determine the reasons for this variation but it may be associated with the general socioeconomic status of the neighbourhoods, as Patel Nagar and Rajinder Nagar are richer than the other areas. As we did not study the sociodemographic profile of the patients visiting the facilities it is not possible to analyse this further. A study conducted in Uttar Pradesh, India showed that higher socioeconomic status of the patients was associated with higher antibiotic use [21].

A small degree of variation was observed in the prescription of different antibiotics over 12 months. In public facilities any variation could have been due to variation in supply of antibiotics to public health facilities from the main central store (though correlation of use with availability was not done). Overall there did appear to be slightly greater use of antibiotics (penicillins, tetracyclines, cotrimoxazole, macrolides) used for acute respiratory tract infections (ARI) in the winter months and for antibiotics used for diarrhea (fluoroquinolones) in the humid summer months. A survey conducted simultaneously with this study showed that number of diarrhea cases did increase during the rainy summer months and that fluoroquinolones were the most commonly prescribed antibiotic for diarrhea [22]. It is likely that much of the antibiotic use was inappropriate particularly with regard to use of fluoroquinolones in the humid summer months for diarrhea. The European Surveillance of Antimicrobial Consumption (ESAC) study has shown a higher outpatient antibiotic use in the winter season in all countries. The authors explained that this seasonal variation could be related to an increased incidence of respiratory tract infections during the winter months in European countries, resulting in higher prescription rates during this period [23]. Acute respiratory infections are more likely in the winter months in Delhi also and it is likely that the slightly higher consumption of some antibiotics during the winter months in our study was due to this and could include inappropriate prescribing for coughs and colds.

The high and increasing use of the newer antibiotics in the community is worrying, as such antibiotics may often not be needed and their use will lead to resistance in the treatment of serious infections. The older antibiotics should be sufficient for many of the common infections seen in the community. While there is a profit-motive in the private sector for using the newer and more expensive antibiotics, the reason for their increased use in the public sector is not clear. There is an essential medicine list (EML) for all public facilities under the Government of National Capital Territory of Delhi, and this list is regularly updated and used for procurement of all medicines. The Delhi state EML has a separate EML for dispensaries and this list has antibiotics, like amoxicillin, ampicillin, ciprofloxacin, norfloxacin, cephalexin (for restricted use), erythromycin, roxithromycin, cotrimoxazole and doxycycline. These antibiotics are the first choice to treat most common infections in the community. Antibiotics like ofloxacin and cefuraxime are added to the hospital list of the EML with an *asterisk indicating that they are reserve antibiotics to be used only in case of significant resistance to other antibiotics [24]. However, our survey revealed that antibiotics for hospital use were available in the dispensaries and were frequently used by the doctors there. A detailed study should be conducted to find out why the newer antibiotics reserved for hospital use are being used in the public dispensaries. It could be due to doctor preference or non-availability of first generation antibiotics. The preference of the private sector to use newer broad-spectrum antibiotics has been seen elsewhere. A study conducted in Italy in primary health care showed [25] that GPs had a tendency to preferentially prescribe wide spectrum antibiotics and to use, in many cases, antibiotics that are rarely of choice in primary health care, such as cephalosporins and fluoroquinolones. Outpatient antibiotic use in 26 countries in Europe has also shown a shift from the old narrow-spectrum antibiotics to the new broad-spectrum antibiotics [11].

Our study has successfully captured the pattern of antibiotic use in the community. We did not measure DDD/1000 inhabitants as is generally done by studies in European countries. In our settings this is not an accurate measure of exposure of the population to antibiotics because we have not included all the facilities of our catchment area and complete data (covering all patient attendees) could not be collected from any enrolled facility. The objective was not to measure accurately population exposure to antibiotics, but rather to measure trends in use as part of a surveillance system. Provided the same methodology is used each month, the same degree of error will occur each month and the data collected will be sufficient for trend measurement even though it is not accurate for a one-off exposure measurement. Since the exit interview data only measures use in some of the patients attending some of the health facilities, the total number of DDDs per population would be much less than would be the case if all the facilities were included.

The most important strength of the study is that it clearly shows that it is possible to collect useful data for antibiotic use in all types of facility from wherever it is prescribed/dispensed/purchased, at the individual patient level in resource-constrained settings. The study has some inherent weaknesses. Firstly, it was conducted in four residential localities of one urban area, so generalization should be done with caution for the rest of Delhi and cannot be done for other areas of India. Secondly, the presence of data collectors may have changed the prescribing and dispensing habits of doctors and retailers. Doctors may have prescribed fewer antibiotics than normal and retailers may not have dispensed antibiotics over-the-counter as often as normal. Some of the Hawthorn effect may have reduced over the period of one year, as the health workers got used to the presence of the data collectors. In public facilities, the bias on doctors' prescribing may have been less as the data collectors were in the pharmacy area and doctors were not always aware which day and time data collectors were visiting. The reduced sample size of exiting patient interviews in private sector clinics, due to time and resource constraints may have resulted in less accurate estimates of antibiotic use in this facility type as compared to the other facility types. However, the year-long duration of the study should partially compensate for the lesser number of patients interviewed each month. While the methodology used is simple, reproducible, and feasible for collecting data from various facilities in the community over a long period of time, it requires attention to detail particularly with regard to supervision of data collection, data management and maintaining good relations with frequent feedback to participating facilities (in order to maintain their cooperation).

## Conclusion

Antibiotic use is the key driver of antimicrobial resistance. Antibiotics are overused, particularly for minor infections, misused for self-limiting viral infections and underused due to financial concerns. Extensive surveillance programmes have been used to study patterns of antibiotic resistance and use in developed countries. These systems have made it possible to stimulate the implementation of nationwide interventions to improve antibiotic use [26]. Results from our study have shown that community-based surveillance of antibiotic use is possible in resource-constrained settings, and patterns of antibiotic use can be surveyed over years. The methodology can collect useful data that reveal trends and patterns of antibiotic use in the community. However, the effort of collecting such data is only worthwhile if policy makers invest in the interventions urgently needed to improve the use of antibiotics in the community and to contain antibiotic resistance. Without such action, increasing antimicrobial resistance will deny future generations the benefit of effective antibiotics to treat common infections.

## Competing interests

The authors declare that they have no competing interests.

## Authors' contributions

Both the authors (AK and KH) are responsible for the reported research, and have participated in the concept and design, analysis and interpretation of data, revising of the manuscript, and have approved the manuscript as submitted. AK was also responsible for getting data collected from different public and private facilities, data entry, and drafting the manuscript.

## Pre-publication history

The pre-publication history for this paper can be accessed here:

http://www.biomedcentral.com/1471-2334/11/99/prepub
